# Acute hepatitis of unknown aetiology among children around the world

**DOI:** 10.1186/s40249-022-01035-2

**Published:** 2022-11-05

**Authors:** Chao Wang, Zhi-Yong Gao, Nick Walsh, Stephen Hadler, Qing-Bin Lu, Fuqiang Cui

**Affiliations:** 1 Department of Laboratorial Science and Technology & Vaccine Research Center, School of Public Health, Peking University, No. 38 Xue-Yuan Road, Haidian District, Beijing, 100191 People’s Republic of China; 2 Global Center for Infectious Disease and Policy Research & Global Health and Infectious Diseases Group, Peking University, Beijing, 100191 People’s Republic of China; 3 Institute for Infectious Disease and Endemic Disease Control, Beijing Center for Disease Prevention and Control and Beijing Research Center for Preventive Medicine, Beijing, People’s Republic of China; 4 Department of Epidemiology and Preventive Medicine, School of Public Health and Preventive Medicine, Monash University, Commerical Road, Melbourne, 3000 Australia; 5 Independent Consultant, Medical Epidemiology, Atlanta, GA USA

**Keywords:** Acute hepatitis, Aetiology, Children, Disease surveillance

## Abstract

By 26 August 2022, the number of cases of acute hepatitis of unknown etiology (AHUA) has drastically increased to 1115 distributed in 35 countries that fulfill the World Health Organization definition. Several hypotheses on the cause of AHUA have been proposed and are being investigated around the world. In the recent United Kingdom (UK) report, human adenovirus (HAdV) with adeno-associated virus (AAV) co-infection is the leading hypothesis. However, there is still limited evidence in establishing the causal relationship between AHUA and any potential aetiology. The leading aetiology continues to be HAdV infection. It is reported that HAdV genomics is not unusual among the population in the UK, especially among AUHA cases. Expanding the surveillance of HAdV and AAV in the population and the environment in the countries with AUHA cases is suggested to be the primary action. Metagenomics should be used in detecting other infectious pathogens on a larger scale, to supplement the detection of viruses in the blood, stool, and liver specimens from AUHA cases. It is useful to develop a consensus-specific case definition of AHUA to better understand the characteristics of these cases globally based on all the collected cases.

## Background

On 5 April 2022, 10 cases of acute hepatitis of unknown aetiology (AHUA) among children aged 11 months to 10 years were first recorded in Scotland with onset from January to March 2022 and reported to the World Health Organization (WHO) [1]. By 26 August 2022, the number of similar probable cases has dramatically increased to 1115 distributed in 35 countries that fulfill the World Health Organization definition [2]. Both Americas and Europe WHO region showed a large increase since their initial reports of the disease among children, in which the United States (US) and the United Kingdom (UK) reported the largest case numbers [2, 3]. Definitions of AHUA across regions or countries have shown slight variations. However, clinical hepatitis symptoms and patients’ age are the most important criteria, along with exclusion of viral hepatitis A-E,  in all definitions (Table 1).

**Table 1:** The latest case definitions of the acute hepatitis of unknown aetiology by different guidelines

Institution	File name	Release date	Confirmed case	Probable case	Epi-linked case	Discarded case/Current patients under investigation
WHO [2]	Severe acute hepatitis of unknown aetiology in children—multi-country	12 July 2022	NA	With an acute hepatitis; None Hep A–E; AST or ALT > 500 IU/L; 16 years and younger; Since 1 October 2021	With an acute hepatitis; None Hep A–E; At any age; Close contact of a probable case; Since 1 October 2021	NA
ECDC [2]	Joint ECDC-WHO Regional Office for Europe Hepatitis of Unknown Origin in Children Surveillance Bulletin, 17 June 2022	29 July 2022	NA	With an acute hepatitis; None Hep A–E; AST or ALT > 500 IU/L; 16 years and younger; Since 1 October 2021	With an acute hepatitis; None Hep A–E; At any age; Close contact of a probable case; Since 1 October 2021	Further investigations did not meet the case definition criteria
UK Health Security Agency [4]	Investigation into acute hepatitis of unknown aetiology in children in England-Technical briefing 3	26 July 2022 England, Wales, Northern Ireland case definitions	With an acute hepatitis; None Hep A–E or an expected presentation of metabolic, inherited or genetic, congenital or mechanical cause; AST or ALT > 500 IU/L; 10 years old and younger; Since 1 January 2022	With an acute hepatitis; None Hep A–E or an expected presentation of metabolic, inherited or genetic, congenital or mechanical cause; AST or ALT > 500 IU/L; 11 to 15 years old; Since 1 January 2022	With an acute hepatitis; None Hep A–E or an expected presentation of metabolic, inherited or genetic, congenital or mechanical cause; Close contact of a confirmed case; Since 1 January 202	NA
UK Health Security Agency [4]		26 July 2022 Scotland case definition	AST or ALT > 500 IU/L without any known cause, 10 years of age and younger or a contact of any age of a confirmed case; Since 1 January 2022 Excluding hepatitis, A–E, cytomegalovirus and Epstein-Barr virus	NA	NA	NA
United States [3]	Technical Report: Acute Hepatitis of Unknown Cause	17 August 2022	NA	NA	NA	10 years of age and younger; AST or ALT > 500 U/L; Have an unknown etiology for hepatitis (with or without any adenovirus testing results, irrespective of the results) Since October 1, 2021

WHO: World Health Organization; AST: Aspartate aminotransferase; ALT: Alanine aminotransferase; UK: United Kingdom; Hep: Hepatitis virus; ECDC: The European Center for Disease Control and Prevention; NA: Not applicable

Of the 1115 cases reported to WHO, 47 (4%) children have required transplants as a result of severe liver failure related to infection, and 22 (2%) deaths have been reported. Of 479 cases with information on gender and age, 48% of cases were male, and most cases (78%) were under 6 years of age [2]. The median number of days from symptom onset to hospitalization was four days according to the 167 cases’ records, with cases experiencing acute hepatitis with significantly increased liver enzyme levels, jaundice, and gastrointestinal symptoms. All the cases showed severe hepatitis symptoms with aspartate aminotransferase (AST) or alanine aminotransferase (ALT) over 500 U/L [1–3]. Cases were sporadically distributed, lacking epidemiological association or travel history. None of the common hepatitis viruses (hepatitis A to E) were detected in these cases, while human adenovirus (HAdV) was detected in at least 209 cases, of which 31 cases with pathogen type detection were identified as HAdV‑F41 from the WHO report, with the most  compelling data from UK or US (Table 2). Severe acute respiratory syndrome coronavirus 2 (SARS-CoV-2) was detected in only 7.7% of cases [2, 4].

**Table 2:** The reported cases of AHUA by countries as of 25, August, 2022

Countries with AHUA reported cases	Cases
Bulgaria	1
Latvia	1
Luxembourg	1
Republic of Moldova	1
Serbia	1
Panama	1
Maldives	1
Occupied Palestinian territories	1
Qatar	1
Cyprus	2
Brazil	2
Colombia	2
Argentina	3
Costa Rica	3
Singapore	3
Israel	5
Austria	6
Norway	6
Denmark	8
France	9
Greece	12
Sweden	12
Belgium	14
Netherlands	15
Poland	18
Indonesia	18
Portugal	20
Canada	21
Ireland	26
Italy	36
Spain	46
Japan	67
Mexico	69
United Kingdom	273
United States of America	358

AHUA: Acute hepatitis of unknown aetiology

## Potential causes or pathogens of AHUA

**Fig. 1:**
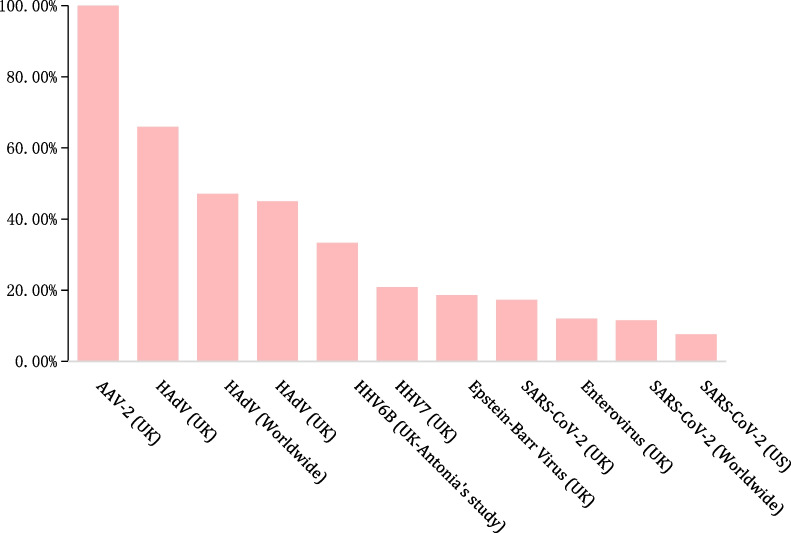
Positive proportion of the potential aetiology in AHUA cases. AHUA: Acute hepatitis of unknown aetiology

Several hypotheses on the cause of AHUA have been proposed and investigated around the world [4]. However, there is still limited evidence in establishing the causal relationship between AHUA and any potential aetiology (Fig. 1).

## Human adenovirus with AAV co-infection is the primary suspected cause of the AHUA

Among the hypotheses, the leading aetiology continues to be HAdV infection, since the overall positive rate of HAdV (47.1%, 209/444) among AHUA cases reported to WHO, largely outweighs other potential aetiologies around the world [2]. For cases in England, by sample type based on the data reported, amongst 258 cases tested for adenovirus, 170 (65.9%) had adenovirus detected [5]. In a UK-wide frequency matched case–control study, multivariable regression analyses with 74 cases and 225 controls indicate that cases have statistically significant higher odds of concomitant HAdV infection compared to controls with an adjusted odds ratio of 35.27 (95% CI 15.23–81.68) [4]. Recent evidence from the UK has indicated a primary association of Adeno-associated virus 2 (AAV2) with AHUA. To present, metagenomics undertaken on blood and liver tissue has detected primarily adeno-associated virus 2 (AAV-2) [5, 6], although AAV is not currently known to cause disease. In the UK study, in 5 cases who underwent liver transplantation, high levels of AAV-2 were detected in the explanted livers. Also, in a case–control investigation of 9 Scottish children hospitalized with AHUA, AAV-2 was identified in the plasma of 9 out of 9 and liver of 4 out of 4 cases, but in 0 out of 13 sera/plasma of age-matched healthy controls, 0 out of 12 children with adenovirus infection without hepatitis and normal liver function, and 0 out of 33 children admitted with hepatitis of other aetiology. This study indicated that 8 out of 9 cases (88.9%) carried the HLA-DRB1*04:01 allele which is also detectable in four out of five liver transplant cases by the UK study.

In comparison, the frequency of HLA-DRB1*04:01 in a control Scottish population (*n* = 974) is 8.9%. Simultaneously, adenovirus (C or F) was found in 6 out of 9 case samples, including 3 out of 4 liver biopsies, while human herpesvirus 6B was detected in 3 out of 9 case samples, including 2 out of 4 liver biopsies, suggesting that AAV2 typically needs a co-infecting ‘helper’ virus for replication, most commonly adenovirus or a herpes virus.

Notably, HAdV may change its original tissue tropism (even the host type) and acquire stronger virulence and transmission capability after genetic recombination. The fowl adenoviruses (FAdVs), especially FAdV-4, cause inclusion body hepatitis in chickens which is characterized by hepatitis. Under the current knowledge, HAdV‑F41 has been rarely reported to cause liver injury in humans, mainly causing diarrhea, nausea, vomiting, and abdominal pain, while FAdV can cause severe liver injury in poultry or birds. Though less likely, we could not deny the potential given the clinical manifestations of these children with AHUA, that HAdV might experience new recombination from FAdV and have spread around the world. This hypothesis may be supported by a higher prevalence of HAdV (mainly F41 type) reported among children with  diarrhea in 2021 [2, 7] and reduced influence of humans on the living environment of birds due to the COVID-19 pandemic. However, it is reported that HAdV genomics is not unusual among the population in the UK, particularly among AHUA cases. Therefore, continuous investigations on the relationship between HAdV and AHUA should be maintained. Epidemological analyses based on the case–control or ecological studies should be used to explore the association of AUHA with HAdV infection.

## SARS-CoV-2 and other pathogens cannot be fully excluded from the cause

SARS-CoV-2 variants have been detected in an only 11.5% (78/611) of biomedical samples from AHUA cases [2]. The possibility of new variants of this virus in resulting the disease is much less likely but still cannot be abandoned. As reported, many AHUA cases with SARS-CoV-2 infection did not provide strong epidemiological evidence on the causal relationship since it was hard to confirm the time interval between SARS-CoV-2 infection and the onset of AHUA [8]. SARS-CoV-2 was not detected by PCR and sequencing in any clinical 207 samples of UK, including liver samples, in cases or controls [5]. Prior exposure to SARS-CoV-2 was similar between AHUA cases and the general children population. Since acute hepatitis has not been a common feature of COVID-19 in children, it bears little chance that SARS-CoV-2 has changed its histotropism in a short time. There is still insufficient evidence to infer that SARS-CoV-2 vaccination leads to AHUA in children. The report of the World Health Organization points out that since the vast majority of AHUA children have not been vaccinated with the SARS-CoV-2 vaccine, the side effects of the SARS-CoV-2 vaccine were not supported as a cause of AHUA at present [2].

Other pathogens, such as Epstein-Barr virus, enterovirus, metapneumovirus, respiratory syncytial virus, and human coronavirus OC43 were detected in a small number of AHUA cases [9]. These pathogens have not been determined as the cause of AHUA cases considering the pathogenicity of the pathogen and the low detection rates.

The causal mechanism for AHUA was still not clarified at present. However, the clinical symptoms of AHUA cases could potentially result from a superantigen-mediated immune-cell activation after virus infection with intestinal trophism in children [10]. Since a low infection rate of SARS-CoV-2 is confirmed in AHUA cases, other viruses like AAV-2 should be investigated to provide evidence of the superantigen mechanism in HAdV sensitized hosts. An emerging novel pathogen might be another aetiology in causing AHUA, especially a novel virus that is not recognized to cause  liver damage. Metagenomics should consistently be used in detecting other infectious pathogens on a larger scale than viruses in the blood, stool, and liver specimens from AUHA cases. More retrospective studies on AHUA should be conducted on the antigen & antibody detection for SARS-CoV-2 and other potential related pathogens among children-patients.

## Toxic agents or foodborne etiology have potential contribution to liver disease

There has been rare evidence indicating AHUA is associated with any potential toxic agents like metals or organic compounds in bio-samples, and the likelihood of the causal role of toxicology has been much downplayed since the UK investigation. However, their “helper” role is still worth further investigation. Since food production is usually a centralized process with a distinct share of a single manufacturer or location, the hypothesis on its relationship with AHUA is somewhat rational that AHUA cases present similar clinical symptoms with similar epidemic characteristics as well. For instance, aflatoxins are confirmed to have strong hepatotoxicity which could cause acute severe hepatic damage among the exposed individuals [5]. Thus environmental or food exposures should be investigated by using epidemiological and laboratory methods to find potential consistent aetiology among AHUA cases.

## Issues to be considered in the future

The accelerated increase in AHUA cases in children reported from 35 countries has aroused substantial concerns within the public. Coordinated efforts should be made to determine the underlying aetiology to support all the following measures in clinical treatment and disease prevention. Based on the current data, most cases are identified in high-income countries or regions whose developed health surveillance systems may act as the key to on-time case detection and validation. This raises an important issue around the capacity to detect and report cases if AHUA cases were to emerge in health resource-limited settings. Thus, surveillance of AHUA cases should be a continuous effort in mapping and updating AHUA epidemic around the world, especially in lower-resourced countries. It should be followed by joint efforts on the detection of the aetiology of AHUA, among which, epidemiology investigations are of priority. Before a certain pathogen is verified, case–control or case-case studies should be maintained and extended to a wider range of the population.

Another important way to offer a clue of the aetiology lies in the measurement of pathological examination of the potential role of immunopathologic damage in the liver. It is rational to test if there is any autoimmune injury after potential virus infection by using detection methods of electron microscopy or immunohistochemical staining.

In addition, we cannot neglect the fact that the current reported AHUA cases might be the tip of the iceberg of the potential aetiology if cases with slight or moderate related diseases were not recorded as AHUA. Current evidence has shown that the HLA-DRB1*04:01 allele was much more prevalent in UK cases [6]. Thus, it is critical to conduct epidemiological investigations around AHUA cases in other areas to determine if the aetiology was also associated with such individual susceptibility differences between AHUA cases and their epi-linked population in resulting clinical symptoms.

## Conclusions

Based on the latest evidence, co-infection of AAV-2 and “helper” virus like HAdV is the primary hypothesis as the cause of AHUA. However, before a certain aetiology is confirmed, any possibility is still worth further verification, by combining epidemiological and laboratory methods in an extended population or sample scale. It is also  urgent to develop a clear and accurate definition of AHUA to better understand the characteristics of these cases globally based on all the collected cases.

## Data Availability

Not applicable. **Ethics approval and consent to participate** Not applicable.

## Abbreviations

AHUA: Acute hepatitis of unknown aetiology
HAdV: Human adenovirus
AAV: Adeno-associated virus AAV
WHO: World Health Organization WHO
AST: Aspartate aminotransferase
ALT: Alanine aminotransferase
SARS-CoV-2: Severe acute respiratory syndrome coronavirus 2
AAV-2: Adeno-associated virus 2
HHV6B: Human herpesvirus 6B
FAdVs: Fowl adenoviruses
US: United States
UK: United Kingdom
Hep: Hepatitis virus
ECDC: The European Center for Disease Control and Prevention
